# Provision of dental prostheses in the oral health care network: a mixed methods study, Goiânia, Brazil, 2019

**DOI:** 10.1590/S2237-96222026v35e20250099.en

**Published:** 2026-03-27

**Authors:** Jéssica Karla Maia Zago, Gabriela Montenegro dos Anjos, Gerald John McKenna, Claudio Rodrigues Leles, Lidia Moraes Ribeiro Jordão

**Affiliations:** 1 Universidade Federal de Goiás, Goiânia, GO, Brazil; 2 Queen’s University Belfast, Belfast, NI, Reino Unido da Grã-Bretanha e Irlanda do Norte

**Keywords:** Primary Health Care, Secondary Health Care, Dental Prosthesis, Health Services, Oral Health, Atención Primaria de Salud, Atención Secundaria de Salud, Prótesis Dental, Servicios de Salud, Salud Bucal

## Abstract

**Objective:**

To analyze the provision of dental prostheses by the oral health care network (OHCN) of Goiânia, capital city of Goiás state, Brazil.

**Methods:**

This was a convergent mixed methods study, with semi-structured interviews, thematic analysis and schematic representation of the OHCN. Monthly data on dental prosthesis placements in 2016 and 2019 were extracted from the Outpatient Care Control System of both the Goiânia City Health Department and the Goiás State Health Department, and their averages were calculated.

**Results:**

22 individuals participated; seven service users, eight dentists from primary health care (PHC), five from dental specialty centers (DSC) and two from oral health administration/appointment coordination. The Goiás state DSC provided an average of 24.5 denture placements/month in 2016, and 27.16 in 2019, while the Goiânia city DSC provided 8.25 placements in 2016 and 2.66 in 2019. The only type of prosthesis provided was the conventional complete denture. Quantitative-qualitative data integration adjustment at the methods level (comparison and merger) confirmed the findings that the OHCN fulfills the requirements for reception in PHC and referral to the DSC, where dental prosthesis procedures were performed. The local network proved slow in providing dental prostheses, whereby meeting prosthetic demands fell short of targets (12.4%).

**Conclusion:**

Despite low productivity, the OHCN ensures conventional complete dentures for service users. According to the interviewees, adequacy of material resources, expansion of monthly appointments for dental prostheses, including in PHC, diversification of other types of prostheses, strengthening the use of counter-referral, and improvement of appointment coordination are measures necessary for improving dental prosthesis provision.

## Resumen

**Objetivo:**

Analizar la provisión de prótesis dentales por la red de atención bucodental (RABD) de Goiânia, estado de Goiás, Brasil.

**Métodos:**

Estudio convergente de métodos mixtos, con entrevistas semiestructuradas, análisis temático y representación esquemática de la RABD. Se extrajeron datos mensuales sobre la colocación de prótesis dentales en 2016 y 2019 del Sistema de Control de Atención Ambulatoria de los departamentos de salud de Goiânia y Goiás en su conjunto, y se calcularon sus promedios.

**Resultados:**

Participaron 22 personas: siete usuarios, ocho odontólogos de atención primaria de salud (APS), cinco de centros de especialidades odontológicas (CEO) y dos de gestión/regulación. En el CEO del estado, se registró un promedio de 24,5 colocaciones de prótesis dentales/mes en 2016 y de 27,16 en 2019. En el sector municipal, hubo 8,25 colocaciones en 2016 y 2,66 en 2019. El único tipo de prótesis proporcionada fue la dentadura completa convencional. El ajuste de la integración de datos cuantitativos y cualitativos a nivel metodológico (comparación y fusión) confirmó que la RABD cumple con los requisitos de atención en la APS y derivación al CEO, donde se realizaron los procedimientos de prótesis dentales. La red local demostró lentitud en la provisión de prótesis dentales, con una resolución de la demanda inferior a los objetivos (12,4%).

**Conclusión:**

A pesar de la baja productividad, la RABD garantiza prótesis dentales completas convencionales para el usuario del servicio. Según los entrevistados, la suficiencia de recursos materiales, la ampliación de las citas mensuales para prótesis dentales, incluso en la APS, la diversificación de otros tipos de prótesis, el fortalecimiento del uso de la contraderivación y la mejora de la gestión de citas son medidas necesarias para mejorar la provisión de prótesis dentales.

**Ethical aspects**

This research respected ethical principles, having obtained the following approval data:

**Research ethics committee**: **Opinion number**, **Approval date**

Universidade Federal de Goiás: 3,060,856, 6/12/2018

Centro de Excelência Leide das Neves Ferreira: 3,064,239, 7/12/2018

**Certificate of submission for ethical appraisal**: 02218618.1.3001.5082; 02218618.1.0000.5083

**Informed consent record**: Obtained from all participants before data collection.

## Introduction 

Severe tooth loss was the 36^th^ most prevalent oral condition in the global population in 2010, being estimated at 2% (1). In Brazil, tooth loss affects adolescents, adults and the elderly, with higher prevalence among the elderly (2). The effects of tooth loss include impaired eating, affected self-confidence and self-image, limitations in speaking and smiling, impaired social interaction, feelings of shame, embarrassment (3) and increased risk of malnutrition (4). In 2023, the proportion of adults (35-44 years) needing dental prostheses was approximately 53% in Brazil, while for the elderly (65-74 years) it was 75% (5).

The way the Brazilian Unified Health System (*Sistema Único de Saúde*, SUS) is organized, with integration of services and actions and articulation between points of health care, seeks to ensure continuous and comprehensive care and to overcome fragmented models (6). Primary care is the communication hub responsible for organizing the care network and coordinating care, while secondary and tertiary care offer specialized services, ensuring that needs are met (6).

Prior to the ordinance that established the guidelines for the organization of the health care network (6), the guidelines of the National Oral Health Policy proposed reorientation of the oral health care model and included: expansion and qualification of primary, secondary and tertiary care; diversification of procedures; care for all age groups; inclusion of prosthetic rehabilitation in primary care; and implementation of Dental Specialty Centers (DSC) as referral units for the provision of services that are specialized and complementary to those of primary oral health teams (7).

Secondary oral health care, provided by DSC, has the following minimum services: oral diagnosis with emphasis on the detection of oral cancer, periodontics, minor oral surgery, endodontics and care for people with special needs (8). Implementation of regional dental prosthesis laboratories intended to offer removable partial and/or total dental prostheses.

Publications concerning the funding of DSC are constantly being updated, due to the need for improvement and expansion of services, which are still offered unevenly across the country (9). Studies on DSC fulfillment of targets (10-13) and production performance and monitoring (14) focused on minimum specialties, in which dental prosthetics is not included.

Primary Health Care (PHC) still follows the principles of the SUS and the care network, which encompass promotion, prevention, diagnosis, treatment, follow-up and rehabilitation actions. Its priority strategy is the consolidation of the Family Health Strategy and it is the preferred entry point for access to the Brazilian health service. It is through this entry point that service users can be referred to other levels of care, being responsible for organizing the flows and counterflows of users, products and information (15).

The updated version of the National Primary Care Policy provides for the responsibilities of PHC dentists, among which is the performance of procedures related to the clinical phases of impression taking, adaptation and follow-up of complete dentures, removable partial dentures and conventional dentures (15). In Brazil, between 2011 and 2014, an increase was observed in the proportion of oral health teams that identified the need for prostheses, as well as in the performance of anatomical and functional impressions (16). Between 2013 and 2014, the frequency of complete denture procedures was 42%, while that of removable partial dentures was 30% (17). The inclusion of dental prostheses in PHC presents challenges, such as the training of dentists to perform procedures and the guarantee of specific supplies. However, PHC is the level of care closest to the population and, due to its capillarity, has the potential for effective universalization of access to this essential service.

There are gaps in the literature regarding the understanding of the different stakeholders in the oral health care network (OHCN) as to the provision of dental prostheses in Goiânia, an important state capital city in the Brazilian Midwest, as well as regarding its actual performance.

The objective of this study was to analyze the provision of dental prostheses by the OHCN in Goiânia in 2019, in order to support actions aligned with the oral health needs of edentulous individuals.

## Methods 

### Design 

This is a cross-sectional observational study that used a convergent mixed-methods approach with simultaneous phases. This study aimed to verify simultaneously quantitative and qualitative information regarding the provision of dental prostheses in the OHCN in Goiânia (18).

### Setting 

In the Goiânia OHCN, in 2019, PHC comprised 41 family health centers and 20 basic health centers spread over seven health districts (North, East, Campinas-Centro, South, Southwest, Northwest and West), with 107 dentists in the basic health centers and 90 in the family health centers. There were five municipally managed DSC with 58 dentists, offering: endodontics, periodontics, maxillofacial surgery, care for people with special needs, stomatology, orthopedics functional jaw, pediatric dentistry and dental prosthetics; and one state-run DSC, which offered the same services as the municipal one plus specialized restorative dentistry and radiology.

In 2007, three prosthetists began working at the municipal DSC, increasing to four in 2024. The state DSC has offered dental prostheses since 2004, with four prosthetists until 2019 and six in 2024. There were two regional dental prosthesis laboratories, one under municipal management and the other state-run.

### Participants 

In the qualitative phase, the key informants were PHC dentists, DSC dentists, municipal oral health administration/appointment coordination dentists, and SUS users. Recruitment was finalized when theoretical saturation was reached. 

The inclusion criteria for service users were: users with SUS appointments; total maxillary and/or mandibular edentulous individuals; and who were at the beginning, in the process of, or at the end of treatment with dental prostheses.

The invitation for service users to participate in the study was made at DSC reception. Following their acceptance, the interviews were conducted individually in a private room. Sociodemographic questions and questions about edentulism, use of dental prostheses, each interviewee’s experiences and expectations about rehabilitation were asked.

The inclusion criteria for dental surgeons were: those currently practicing at the time of data collection and with a minimum of six months of experience in that position. The exclusion criterion was communication difficulties. With regard to the central-level managers, both of them were included, one of them being an oral health service administrator and the other a dentist responsible for appointment coordination.

### Qualitative phase data collection and analysis

Six PHC centers in the Campinas-Centro and East health districts (three family health centers and three basic health centers) were randomly selected. Two DSC with dental prosthetics and the municipal Oral Health Administration were also selected. Both districts were selected by convenience.

The invitation for the health centers to participate was made by telephone, at which time information about the research was provided. After authorization from the health center managers and confirmation of acceptance by the dental professionals working there, dates and times were scheduled for data collection.

The semi-structured interviews were conducted in a spoken and face-to-face manner, individually with PHC and DSC professionals, and in a group interview with those from oral health administration/appointment coordination, by a trained researcher, who was a female dental surgeon with a master’s degree in dentistry and experience in research.

The interview scripts for dental professionals were customized by area of ​​expertise and included professional profile, perception of the care network, administration, treatments available, criteria observed for referral and counter-referral. The scripts were developed by the team with questions that covered the study objectives based on the National Program for Improving Dental Specialty Centers Access and Quality (19) and on a study conducted in Greater Natal (20).

The interviews took place between March and July 2019, and were recorded using a Sony ICD-PX240 digital voice recorder and subsequently transcribed. Thematic content analysis was conducted (21): pre-analysis, material exploration and result treatment/interpretation. The sense units were determined by two independent researchers, and similar codes were grouped, classified, compared and discussed. The initial categories that emerged were revised and refined.

### Data sources and measurement, and statistical methods

For the quantitative phase, data were extracted from the Outpatient Care Control System of the Goiânia Municipal Health Department and the Goiás State Health Department. A descriptive analysis was made of the number of dental prosthesis placements performed by the DSC in 2016 and 2019. The time frame is justified because, in the period 2017-2018, there was service interruption in the DSC due to renovations and relocation.

The data were tabulated using Microsoft Excel 2019 and presented in absolute frequencies, including calculation of the monthly average. The average number of dental prosthesis placements was compared with local targets, informed by local oral health administration and the Ministry of Health (22).

### Study phase integration

The data integration adjustment was used to determine confirmation, expansion or disagreement between the findings. The data were analyzed separately and subsequently combined for comparison and merging, performing integration at the methods level. Integration at the interpretation and reporting level was performed using a contiguous narrative approach, in which the description of the results was presented in a single report (18).

## Results 

Twenty-two participants were interviewed: eight primary health care dentists (five from family health centers and three from basic health centers), five dentists from dental specialty centers (DSC), two from oral health administration/appointment coordination, and seven service users.

The average age of the dentists (n=15) was 45.6 years. Those working in PHC were primarily women (n=7), while those working in the DSC were primarily men (n=3). At both levels, 100% were specialists and had been hired by means of a competitive entrance examination. The majority had graduated from public higher education institutions (six from PHC, four from DSC). Time since graduation ranged from 16 to 33 years. Most dentists in PHC and DSC had been working in the SUS for between 11 and 20 years (n=10). The two participants who worked in oral health administration were women and had been in their positions for between one and three years.

The users were mostly women (n=5), retired (n=4), married (n=4), with a monthly family income of up to three minimum wages (n=6), with an average age of 68.2 years and 0-8 years of schooling (n=3) and 9-11 years of schooling (n=3). One participant did not provide information. Most used prostheses in both arches (n=4), two used them only in the maxillary arch, and one did not use any. Time since total edentulism was 0-20 years in both arches (n=3), ≥45 years for the maxillary arch (n=3), and one user was unable to answer. With regard to the mandibular arch, most were partially edentulous (n=4). Waiting time with effect from referral was >12 and ≤24 months (n=3), >24 and ≤36 months (n=2) and 0-12 months (n=2).

**Figure 1:**
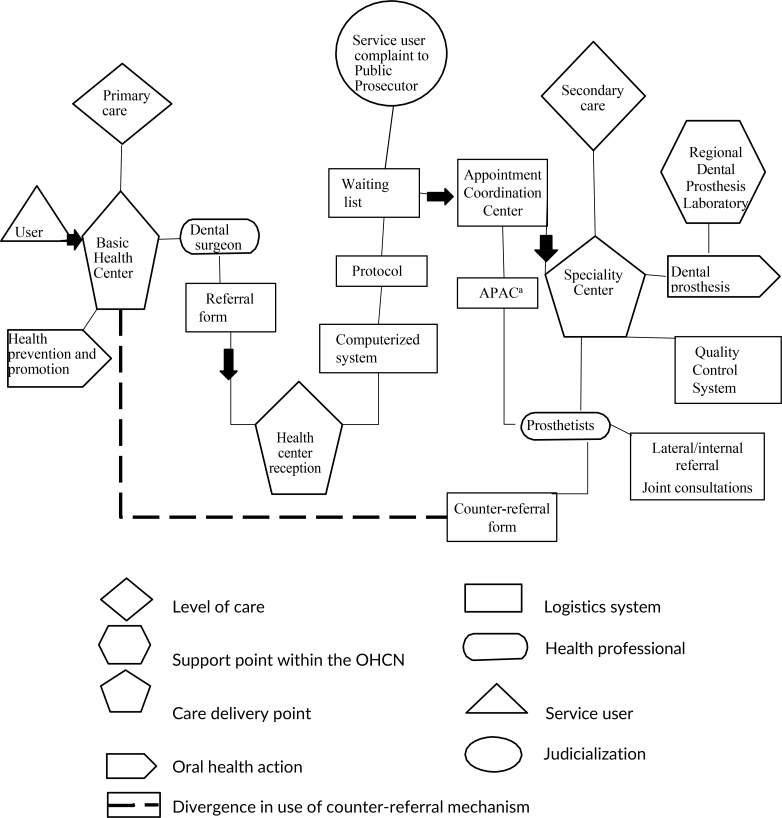
Representation of the oral health care network (OHCN) for treatment with dental prosthesis, according to dentist and service user statements. Goiânia, 2019 (n=22)

We analyzed the constituent elements of the oral health care network in Goiânia for dental prosthesis treatment (Figure 1). The service user (triangle) follows a path (arrows along the line) from entering the health service to placement of the complete prosthesis at the DSC. When the PHC dentist needs to refer the service user for dental prosthesis treatment, they fill out the referral form and give it to the user, who then goes to the reception area, where the form is registered on the computerized system. The Centralized Appointment System coordinates the waiting list for the specialty and for the service user. Once selected, the user is scheduled for treatment at the DSC.

Regarding this process, it was reported that service users filed complaints with the Public Prosecutor’s Office in order to expedite the process. After receiving the service user at the DSC, the prosthetist completes online the examination report for authorization of high-cost/complex procedures in order to justify the prosthesis request. Regional dental prosthesis laboratories are support units for prosthesis production. At the state DSC, there is an assessment and quality service that investigates the user’s needs and refers them to another specialty if necessary. The counter-referral form is completed at the end of treatment.

The categories that emerged from the statements of the PHC and DSC dentists were organized into common and exclusive categories (Table 1). Regarding the functioning of the referral and counter-referral system (category 1), the service user is registered on the computerized system and referred to the DSC. Some refer cases that they consider urgent with priority, but are unaware if they are in fact prioritized.

**Table 1:** Common and exclusive thematic analysis categories and subcategories: statements by dentists in Primary Health Care (PHC) and Dental Specialty Centers (DSC). Goiânia, 2019 (n=13)

Categories	Subcategories	Main statements
**Common categories and subcategories**		
1. How the Referral and Counter-Referral System Works	1.1 Service user referral from PHC to DSC	1.1 “Patients have their appointments coordinated by the City Health Department, via the basic health centers to the Specialty Center.” (Dentist 1 – DSC).
	1.2 Attempt to prioritize certain cases	1.2 “[...] a patient who uses a prosthesis is traumatized and will not want to be without a prosthesis. This patient is a priority, because he needs it and because he’s feeling hurt and we request urgency, but I don’t know if it makes any difference.” (Dentist 5 – PHC).
2. Communication between primary and secondary care dentists	2.1 Feedback on the counter-referral and divergences in the use of this mechanism	2.1 “[...] the greatest difficulty we have is to know whether the referral we make results in something, because we’ve never had a patient who returned [...]” (Dentist 6 – PHC). 2.1 “Whenever I finish a treatment, I request counter-referral to primary care” (Dentist 2 – DSC).
	2.2 Importance of communication/interaction between professionals	2.2 “Undoubtedly [it would be interesting to keep in contact], if only for us to monitor the progress of this patient’s treatment.” (Dentist 1 – PHC).
3. Perception as to resolution of prosthetic demands	3.1 Prosthetic treatment alternatives are limited at the DSC	3.1 “There should be an increase in the physical size of the DSC, the number of people it cares for, offer treatment with dental implants, fixed prosthesis, of the metal/ceramic type, for example, that we don’t have here.” (Dentist 3 – DSC).
	3.2 DSC with limited resources/congestion	3.2 “[...] There should be an increase in the number of laboratories, dentists who provide care, prosthetists, capacity is still low in view of the large demand that exists.” (Dentist 5 – PHC). 3.2 “Patients wait for a long, long time. It’s not a matter of six months, it’s years.” (Dentist 2 – PHC). 3.2 “[...] we would have greater patient demand if we had more professionals, more instruments, more materials, more laboratory technicians.” (Dentist 2 – DSC).
	3.3 Service user satisfaction/patients’ needs met/treatment availability	3.3 “[...] for the most part, patients who receive prosthesis leave here satisfied.” (Dentist 4 – DSC).
	3.4 Complexity of the patient’s journey between the OHCN points	3.4 “The greatest difficulty is patients managing to get here because of this trek, having to be seen at a basic health center, before being able to be seen at a DSC [...]” (Dentist 3 – DSC).
4. Role of the appointment coordination service	4.1 Appointment coordination and allocation	4.1 “Essential. It is through appointment coordination that we adopt the criterion of fairness and avoid any other type of situation other than that of a patient with a coordinated appointment.” (Dentist 5 – DSC).
	4.2 Difficulty in understanding how appointment coordination works	4.2 “[...] what I don’t know is how appointment coordination works.” (Dentist 3 – PHC).
**Exclusive categories and subcategories**		
5. Perception as to the role of PHC (dentist – PHC)	5.1 Responsible for service user flow	5.1 “[...] a patient arrives, we identify the problem and refer them if necessary, and we do what we are able to resolve here.” (Dentist 4).
	5.2 Provision of oral health care	5.2 “[...] we give guidance on looking after the prosthesis, sanitizing it, taking care of the prosthesis, in terms of oral health prevention and promotion [...]” (Dentist 1).
6. DSCs in the context of the network (dentist – DSC)	6.1 Specialist professionals trained and integrated with the other specialties	6.1 “We all do refresher courses. We attempt to dedicate ourselves a lot, to study and correct certain shortcomings. To talk with other professionals, interact with other specialties.” (Dentist 2).
	6.2 Positive effects of treatment with total conventional prosthesis	6.2 “[...] when we manage to ask the patient to come, care for them and finish the treatment, it’s always very positive, because we manage to give the patient renewed quality of life.” (Dentist 5).

Dentists considered how communication between themselves (category 2) would be important for monitoring the service user’s journey through the network, mentioned the absence of counter-referral from the dental prosthesis specialty to PHC and how this would contribute to the acquisition of information. The professionals demonstrated their perceptions of the factors that favor and hinder the meeting of prosthetic demands in the network (category 3): restricted alternatives for prosthetic treatment, insufficient material resources (equipment, supplies and instruments), in addition to excessive demand, which constitute congestion in the DSC.

Even when oral health conditions were unfavorable, such as in alveolar bone resorption, dentists demonstrated belief in resolving the needs of edentulous patients. Patient satisfaction with treatment at the DSC and with the assurance of conventional complete dentures was reported.

In category 4, there was heterogeneous understanding of the role of the appointment coordination service in managing appointments and matching service users; some considered it important for the organization of the system, while others appeared unaware. In category 5 (exclusive to PHC), professionals agreed that PHC fulfills the responsibility for user flow, carrying out reception, evaluation and referral to DSC, in addition to assistance in health prevention actions. Positive aspects of treatment at the DSC (category 6 – exclusive to dentists at the centers) were listed, such as qualified professionals and the availability of conventional complete dentures.

We analyzed the categories and subcategories of the dentists’ statements regarding oral health administration/appointment coordination (Table 2). For them, appointment coordination is important for the network (category 1), with its impartial allocation of appointments, following the order of the waiting list and individual analysis of cases, which can be considered priorities.

**Table 2:** Thematic analysis categories and subcategories: oral health administration/appointment coordination dentists. Goiânia, 2019 (n=2)

Categories	Subcategories	Main statements
**1. Work of the appointment coordination service**	None	1.1 “We analyze them case by case (the History of the Current Disease of each patient). Those who are priority will have the first available appointments, those who are not go the end of the waiting list and priority is given to those who have been patients for longer.” (Dentist 1 – administration/appointment coordination).
**2. Oral health administration at the network’s central leve**	2.1 Municipality’s intention to expand dental prosthesis alternatives	2.1 “We have projects for providing removable partial prostheses as well.” (Dentist 2 – administration/appointment coordination).
	2.2 Use of specialized oral health care slots by other municipalities	2.2 “It comes specified, two appointments for the state interior and five for Goiânia [...]” (Dentist 2 – administration/appointment coordination).
**3. Recognition of the network’s limitations**	3.1 Primary care overload	3.1 “Demand is high because availability is infinitely below what patients need.” (Dentist 1 – administration/appointment coordination).
	3.2 Difficulties in prioritizing service users	3.2 “This was one of the difficulties we identified here: prioritizing patients.” (Dentist 1 – administration/appointment coordination).
	3.3 Partial presence of oral health teams in the Family Health Strategy	3.3 “[...] there still are not oral health teams in 100% of the Family Health teams.” (Dentist 2 – administration/appointment coordination).

The oral health administrator demonstrated an intention to expand rehabilitation modalities, provided that the structure available operates at full capacity (category 2). Oral Health Administration has an administrative relationship with municipal health departments in the state of Goiás, which influences the distribution of DSC slots. Limiting factors (category 3) were: the demand for PHC exceeds the supply capacity; difficulties in establishing prioritization parameters for dental prostheses; and access to oral health in the Family Health Strategy is only partially implemented.

We analyzed the categories and subcategories of the service users’ statements (Table 3). Of all those referred by PHC, two filed complaints with the Public Prosecutor’s Office (category 1). Most mentioned delays in accessing the DSC, but satisfaction with the service. One participant demonstrated understanding of the high demand and waiting time (category 2). There was an expectation of greater satisfaction with the new prosthesis than with the current one, as well as intra- and interpersonal effects on regaining a smile, self-esteem and better social interaction (category 3).

**Table 3:** Thematic analysis categories and subcategories: service user statements. Goiânia, 2019 (n=7)

Categories	Subcategories	Main statements
**1. Means of access to Dental Specialty Centers (DSC)**	1.1 Service users referred to DSCs via primary care	1.1 “I came from the Integrated Health Care Service (CAIS), I was referred here.” (Service user 3).
	1.2 Complaint filed with the Public Prosecutor’s Office	1.2 “[...] I had to resort to the Public Prosecutor’s Office to get seen here; otherwise I’d still be waiting at home today without a single tooth.” (Service user 1).
**2. Perspective of waiting time to receive care at a DSC**	2.1 Delay in getting an appointment is outweighed by satisfaction with the service	2.1 “I faced [difficulties], because of the delay. [...] But I was really pleased when they asked me to come.” (Service user 5). “The demand is very high. So there really has to be a waiting list [...]” (Service user 5).
**3. Service user expectation**	3.1 Positive expectation	3.1 “I hope that [the prosthesis] will be better. Because this [prosthesis] that I’m [...] is broken. So I’m sure that it will improve greatly.” (Service user 5).
	3.2 Intra- and interpersonal repercussions	3.2 “[...] to be able to smile, look in the mirror and give a smile and resume life, self-esteem [...]. I will be able to frequent certain environments, [...] because depending on the type of social interaction, I wouldn’t even want to participate in some environments [...]” (Service user 1).
**4. Perception regarding satisfaction**	4.1 Satisfaction with care received and recommendation of the DSC	4.1 “I recommend [the DSC]. The service was good, the staff were competent, and I liked it. I would recommend it.” (Service user 2).
	4.2 (Dis)satisfaction with conventional complete prosthesis	4.2 “People say that lower dentures are very difficult to get used to, it’s more or less to be expected. There are times when you want to talk, want to go out, and you have to get on with it.” (Service user 2). “Everything was great. My new tooth is wonderful.” (Service user 6).
	4.3 Dissatisfaction with lack of alternatives	4.3 “It was my dream to have a small implant, a prosthesis with four... at least four. Two at the front and two further back to hold it properly, but so far it’s not available.” (Service user 2).
**5. Importance of the Brazilian Unified Health System (Sistema Único de Saúde, SUS)**	5.1 Dependence on the SUS	5.1 “[If the DSC didn’t exist] I had no way of doing anything. [...] I have a denture but it’s very old, I’ve had it for fifty years.” (Service user 6).
	5.2 Notion of the right to health	5.2 “[If the DSC didn’t exist] I would probably appeal everywhere... after I looked into all of this, I went to the Public Prosecutor’s Office, and from there, my inclination was to knock on the doors of the press, the news network.” (Service user 1).

Service users expressed satisfaction and said they would recommend the DSC. There was partial satisfaction with retention of mandibular prostheses and dissatisfaction with the lack of treatment alternatives, such as dental implants and removable partial dentures (category 4).

The importance of the SUS in restoring health and the right to health was noted (category 5). Some would not be able to afford to pay for a prosthesis, while others would seek private services when possible. Two participants appeared to be aware of the right to health guaranteed by law when they stated that they would seek assistance from the Public Prosecutor’s Office in the absence of a DSC.

At the state-level DSC, an average of 24.5 dental prosthesis placements were performed per month in 2016 and 27.16 in 2019. In both years, four prosthetists were working at the DSC (20 hours/week). The target was 120 prosthesis placements per month for that number of professionals. However, only 22.6% of the target was achieved in 2019.

At the municipal DSC, the monthly average was 8.25 dental prosthesis placements in 2016 and 2.66 in 2019. In 2016, there were five prosthetists working, while in 2019 there were four (20 hours/week). The daily target per dentist was five patients, one examination (new patient) and four treatments (return visits). In 2019, only 2.21% of the target was achieved.

There was confirmation between the phases of the research when data integration adjustment was performed. It was found that the network fulfills the role of receiving service users in PHC and referring them to specialized care, but that the network is congested regarding dental prosthesis treatment. Unavailability of material resources, scarcity of prosthesis alternatives, and slowness in accessing a DSC contributed to the low resolution of prosthetic demands, in line with the performance below the targets for dental prosthesis placements.

## Discussion 

This study highlighted weaknesses in the operational structure of the oral health care network in Goiânia, especially in the provision and production of dental prostheses. The perceptions of service users and oral health professionals pointed to limitations in access, effectiveness and meeting targets, particularly in SUS specialized care.

Law No. 14572/2023, which established the SUS National Oral Health Policy, strengthens the right to universal, equitable and continuous access to oral health services. PHC plays a central role in this process, being responsible for receiving, assessing and referring patients to the specialized level, as also provided for by the National Primary Care Policy (15) and by the comprehensive care guidelines. Even so, provision of dental prostheses in PHC remains restricted, despite there being teams to do this.

In this study, it was found that PHC receives service users, performs oral health assessment, provides care and referral to the specialized level of the SUS, in addition to prevention actions (15). Service user reception is related to longitudinality and satisfaction in PHC (23).

Only part of the teams perform prosthetic procedures, and coverage is still insufficient (11,24). In the Midwest region of Brazil, where Goiânia is located, there was a decrease in impression taking and follow-up appointments (16), indicating backsliding in the provision of these services. Although dentists can perform dental prosthetic procedures in primary health care, not all oral health teams in Brazil provide them (24), as in this study, where the provision of prostheses takes place in secondary care. The qualitative phase of a study conducted with teams from Belo Horizonte in 2019 (24) found that provision of prostheses and diagnosis of oral cancer influence their performance (24).

Provision of prosthetic procedures, including removable partial dentures, needs to be integrated more into the work processes of PHC teams. Despite the challenges, this would contribute to health care comprehensiveness (25).

Oral health coverage in the Family Health Strategy was 31% in Goiânia, this being below the desired level. Absence of counter-referral – important for articulation between the levels of care in the SUS – is also critical, with the Midwest region presenting the worst rates in the country (26).

The waiting time for specialized care is long, especially for dental prostheses, which discourages service users and compromises care. Although the DSC have prosthetists, productivity is below expected, often due to lack of supplies, equipment or demand overload. The production targets for complete dentures have not been met, even with established clinical and laboratory care parameters (22).

Low achievement of DSC targets for endodontics was found in the Southern region of Brazil (12). In the state of Maranhão, a study found that most DSC did not meet the targets for endodontics, oral surgery and periodontics (13). Long waiting lists, impaired geographical accessibility and lack of materials were negative aspects reported by service users regarding the effectiveness of health services (27). In Brazil, in 2012, the reasons for DSC service shortfalls involved the lack of functioning equipment, supplies and instruments (26), which reinforces the need for regular maintenance and successful purchasing processes.

Tooth loss without rehabilitation directly affects an individual’s quality of life (3). Fortunately, service users demonstrated satisfaction with the care received at the DSC and reported positive expectations regarding dental prostheses, mainly in the recovery of self-esteem and functionality, findings similar to the investigation in which users expressed satisfaction with DSC (29).

Despite the limitations of this study – such as conducting the interviews with users in a single DSC and a possible gratitude bias – the findings contribute to the planning and improvement of the oral health care network in Goiânia. Recommendations include strengthening the provision of prostheses in the DSC, including provision of other types of prostheses, expanding the number of monthly appointments in the DSC, adjusting the number of prosthetists and material resources, as well as improving management of the waiting list and the referral/counter-referral system.

We highlight that complete dentures are provided by the network, with users’ needs met in PHC and appropriate referral to a DSC, where professionals perform available treatment whenever possible. Service users expressed satisfaction and pointed to positive expectations regarding dental prostheses, including improvements in self-esteem, interpersonal relationships and functional activities.

The effective implementation of the National Oral Health Policy requires coordination between levels of healthcare and the guarantee of adequate infrastructure to ensure comprehensive care for SUS service users. 

## Data Availability

The data used in the research are available upon request to the authors, via the email provided in the Correspondence section of this article.
