# Supplementary material for: Provision of dental prostheses in the oral health care network: a mixed methods study, Goiânia, Brazil, 2019
Source: Epidemiol Serv Saude. 2026 Mar 27;35:e20250099. doi: 10.1590/S2237-96222026v35e20250099.en (PMC13030123; doi:10.1590/S2237-96222026v35e20250099.en)
Supplement: Translation

# Translation: A rede de atenção à saúde bucal na provisão de prótese dentária: estudo de métodos mistos, Goiânia, 2019

**DOI:** 10.1590/S2237-96222026v35e20250099.pt

**Authors:** Jéssica Karla Maia Zago, Gabriela Montenegro dos Anjos, Gerald John McKenna, Claudio Rodrigues Leles, Lidia Moraes Ribeiro Jordão

**Affiliations:** 1 Universidade Federal de Goiás, Goiânia, GO, Brasil; 2 Queen’s University Belfast, Belfast, NI, Reino Unido da Grã-Bretanha e Irlanda do Norte

**Keywords:** Atenção Primária à Saúde, Atenção Secundária à Saúde, Prótese Dentária, Serviços de Saúde, Saúde Bucal

## Abstract

**Objetivo:**

Analisar a provisão de prótese dentária pela rede de atenção à saúde bucal (RASB) de Goiânia, Goiás.

**Métodos:**

Estudo de métodos mistos convergente, com entrevistas semiestruturadas, análise temática e representação esquemática da RASB. Os dados mensais de instalações de prótese dentária em 2016 e 2019 foram extraídos do Sistema de Controle do Atendimento Ambulatorial das secretarias de saúde de Goiânia e Goiás como um todo, tendo as suas médias calculadas.

**Resultados:**

Participaram 22 indivíduos; sete usuários, oito cirurgiões-dentistas da atenção primária à saúde (APS), cinco dos centros de especialidades odontológicas (CEO) e dois da gestão/regulação. No CEO estadual, ocorreram em média 24,5 instalações de prótese dentária/mês em 2016, e 27,16 em 2019; no municipal, 8,25 instalações em 2016 e 2,66 em 2019. O único tipo de prótese fornecido foi a total convencional. O ajuste da integração de dados quanti-qualitativos no nível métodos (comparação e fusão) confirmou os achados de que a RASB cumpre com o acolhimento na APS e o encaminhamento ao CEO, onde procedimentos para prótese dentária foram realizados. A rede local apresentou-se morosa na provisão de prótese dentária, com resolutividade das demandas protéticas aquém das metas (12,4%).

**Conclusão:**

Apesar da baixa produtividade, a RASB assegura a prótese total convencional ao usuário. A adequação dos recursos materiais, a expansão de vagas mensais para a prótese dentária, inclusive na APS, a diversificação de outros tipos de prótese, o reforço na utilização da contrarreferência e o aprimoramento do gerenciamento de vagas são, de acordo com as partes entrevistadas, medidas necessárias para aprimorar a provisão.

**Aspectos éticos**

A presente pesquisa respeitou os princípios éticos, obtendo os seguintes dados de aprovação: :

**Comitês de ética em pesquisa**: **Números dos pareceres**, **Datas de aprovação**

Universidade Federal de Goiás: 3.060.856, 6/12/2018

Centro de Excelência Leide das Neves Ferreira: 3.064.239, 7/12/2018

**Certificado de apresentação de apreciação ética**: 02218618.1.3001.5082; 02218618.1.0000.508

**Registro do consentimento livre e esclarecido**: Obtido de todos os participantes antes da coleta.

## Introdução 

A perda dentária severa foi a 36ª condição mais prevalente na população mundial em 2010, com estimativa de 2% (1). No Brasil, a perda dentária acomete adolescentes, adultos e idosos, havendo maior prevalência entre idosos (2). Os efeitos da perda dentária incluem alimentação prejudicada, autoconfiança e autoimagem afetadas, limitações ao falar e ao sorrir, prejuízos no convívio social, sentimentos de vergonha, constrangimento (3) e maior risco de desnutrição (4). Em 2023, a proporção de adultos (35-44 anos) com necessidade de prótese dentária era de aproximadamente 53% no Brasil, já de idosos (65-74 anos) era de 75% (5). 

A organização do Sistema Único de Saúde (SUS), com integração de serviços e de ações e com articulação entre os pontos de atenção à saúde, busca assegurar o cuidado contínuo e integral e superar modelos fragmentados (6). A atenção primária é o centro de comunicação responsável pela organização da rede e pela coordenação do cuidado, enquanto a secundária e a terciária ofertam serviços especializados, garantindo que as necessidades sejam atendidas (6).

Previamente à portaria que estabeleceu as orientações para a organização da rede (6), as diretrizes da Política Nacional de Saúde Bucal propuseram reorientar o modelo de atenção em saúde bucal e incluíam: ampliação e qualificação das atenções primária, secundária e terciária; diversificação de procedimentos; atendimento a todos os grupos etários; inserção da reabilitação protética na atenção primária; e implantação dos centros de especialidades odontológicas (CEO) como unidades de referência para a oferta de serviços especializados e complementares aos das equipes de saúde bucal (7). 

A atenção secundária em saúde bucal, representada pelos CEO, tem como serviços mínimos: diagnóstico bucal com ênfase na detecção do câncer bucal, periodontia, cirurgia oral menor, endodontia e atendimento a pessoas com necessidades especiais (8). A implantação dos laboratórios regionais de próteses dentárias visou ofertar prótese dentária parcial removível e/ou total. 

Publicações que dispõem sobre o financiamento dos CEO estão em constantes atualizações, devido à necessidade de aprimoramento e à ampliação dos serviços, que são ainda ofertados de forma desigual no país (9). Estudos sobre o cumprimento de metas (10-13), o desempenho e o monitoramento de produção dos CEO (14) focaram as especialidades mínimas, nas quais a prótese dentária não está incluída. 

A atenção primária à saúde (APS) segue ainda os princípios do SUS e da rede de atenção, os abrangem ações de promoção, prevenção, diagnóstico, tratamento, acompanhamento e reabilitação. Ela tem como estratégia prioritária a consolidação da Estratégia Saúde da Família e é a porta de entrada preferencial para acesso ao serviço de saúde brasileiro. É através dela que os usuários podem ser referenciados aos demais níveis, sendo a responsável por ordenar os fluxos e os contrafluxos de usuários, produtos e informações (15).

Com a atualização da Política Nacional de Atenção Básica, estão previstas as atribuições do cirurgião-dentista da APS, entre as quais consta a de realizar procedimentos relacionados às fases clínicas de moldagem, adaptação e acompanhamento de prótese total, parcial removível e elementar (15). No Brasil, entre 2011 e 2014, observou-se aumento na proporção de equipes de saúde bucal que identificaram a necessidade de prótese, bem como na realização de moldagem anatômica e funcional (16). Entre 2013 e 2014, a frequência de procedimentos de prótese total foi 42%, e a de prótese parcial removível, 30% (17). A inserção da prótese dentária na APS apresenta desafios, tais como a capacitação dos cirurgiões-dentistas para a realização de procedimentos e a garantia de insumos específicos. No entanto, a APS é o nível de atenção mais próximo às pessoas e, devido à sua capilaridade, apresenta potencial para a efetiva universalização do acesso a esse serviço tão essencial.

Observam-se lacunas na literatura sobre a compreensão das diferentes partes interessadas da rede de atenção à saúde bucal (RASB) na provisão de prótese dentária em Goiânia, importante capital do Centro-Oeste brasileiro, bem como sobre seu real desempenho.

Este estudo teve por objetivo analisar a RASB de Goiânia, em 2019, na provisão de prótese dentária, a fim de subsidiar ações alinhadas às necessidades em saúde bucal da pessoa desdentada.

## Métodos 

### Delineamento 

Trata-se de estudo observacional transversal que utilizou método misto convergente de fases simultâneas. Este estudo visou verificar concomitantemente as informações quantitativas e as qualitativas no que se refere à provisão de prótese dentária na RASB de Goiânia (18).

### Contexto 

Na RASB de Goiânia, em 2019, a APS contava com 41 centros de saúde da família e 20 centros de saúde em sete distritos sanitários (Norte, Leste, Campinas-Centro, Sul, Sudoeste, Noroeste e Oeste), além de 107 cirurgiões-dentistas nos centros de saúde e 90 nos centros de saúde da família. Havia cinco CEO de gestão municipal com 58 dentistas, os quais ofertavam: endodontia, periodontia, cirurgia bucomaxilofacial, atendimento a pessoas com necessidades especiais, estomatologia, ortopedia funcional dos maxilares, odontopediatria e prótese dentária; e um CEO estadual, que ofertava os mesmos serviços do municipal acrescido da dentística especializada e da radiologia. 

Em 2007, três protesistas começaram a atuar no CEO municipal, sendo quatro em 2024. O CEO estadual oferta prótese dentária desde 2004, com quatro protesistas até 2019 e, em 2024, com seis. Havia dois laboratórios regionais de prótese dentária, um de gestão municipal e outro estadual.

### Participantes 

Na fase qualitativa, os informantes-chave foram cirurgiões-dentistas da APS, do CEO, da gestão/regulação municipal e usuários do SUS. O recrutamento foi finalizado com a saturação teórica.

Os critérios de inclusão quanto aos usuários foram: usuários regulados pelo SUS; desdentados totais maxilar e/ou mandibular; e no início, durante ou final do tratamento com prótese dentária. 

Os usuários foram convidados na recepção do CEO. Após o aceite, as entrevistas foram conduzidas individualmente em sala reservada. Abordaram-se questões sociodemográficas e sobre o edentulismo, o uso de prótese dentária, as experiências de cada um e as suas expectativas para a reabilitação. 

Os critérios de inclusão para os cirurgiões-dentistas foram: em exercício no momento da coleta e com experiência mínima de seis meses na função. O critério de exclusão foi dificuldades na comunicação. Para as gestoras do nível central, ambas que atuavam como gerente e cirurgiã-dentista reguladora foram incluídas.

### Coleta e análise de dados da fase qualitativa

Seis unidades de APS dos distritos sanitários Campinas-Centro e Leste (três centros de saúde da família e três centros de saúde) foram sorteadas. Dois CEO com prótese dentária e a Gerência de Saúde Bucal municipal foram selecionados. Ambos os distritos foram selecionados por conveniência.

O convite às unidades aconteceu por telefone, ocasião na qual se forneceram informações sobre a pesquisa. Após a autorização dos gestores e a confirmação do aceite pelos profissionais, agendaram-se os horários para a coleta. 

As entrevistas semiestruturadas foram conduzidas em diálogo e face a face, individualmente com os profissionais da APS e dos CEO, e em grupo com os da regulação/gestão, por uma pesquisadora treinada, cirurgiã-dentista, mestra em odontologia e com experiência em pesquisa . 

Os roteiros para os profissionais foram customizados por atuação e incluíram perfil profissiográfico, percepção sobre a rede, gestão, tratamentos disponíveis, critérios observados para referência e contrarreferência. Eles foram elaborados pela equipe com perguntas que contemplavam os objetivos do estudo baseadas no Programa Nacional de Melhoria do Acesso e da Qualidade dos Centros de Especialidades Odontológicas (PMAQ-CEO) (19) e em estudo da grande Natal (20) (Material Suplementar).

As entrevistas ocorreram entre março e julho de 2019, sendo registradas por gravador de voz digital Sony ICD-PX240 e, posteriormente, transcritas. Conduziu-se análise de conteúdo de modalidade temática (21): pré-análise, exploração do material e tratamento/interpretação dos resultados. As unidades de registro foram determinadas por duas pesquisadoras independentes, e os códigos similares foram agrupados, classificados, comparados e discutidos. As categorias iniciais que emergiram foram revisadas e refinadas.

### Fontes de dados, mensuração e métodos estatísticos 

Para a fase quantitativa, extraíram-se os dados do Sistema de Controle do Atendimento Ambulatorial das Secretarias de Saúde de Goiânia e de Goiás. Realizou-se análise descritiva do número de instalações de prótese dentária pelos CEO em 2016 e em 2019. O recorte temporal se justifica, já que, no período 2017-2018, houve interrupção dos atendimentos nos CEO por motivos de reforma e de mudança de local. 

Os dados foram tabulados no Microsoft Excel 2019 e apresentados em frequência absoluta, sendo a média mensal calculada. A média de instalações de prótese dentária foi comparada com as metas locais, informadas pela gestão local e do Ministério da Saúde (22).

### Integração das fases do estudo

O ajuste da integração de dados foi utilizado para determinar confirmação, expansão ou discordância entre os achados. Os dados foram analisados de forma separada e, posteriormente, reunidos para comparação e fusão, efetuando-se a integração no nível dos métodos. A integração no nível de interpretação e de relatório foi realizada por meio de narrativa com abordagem contígua, em que a descrição dos resultados foi em um único relatório (18).

## Resultados 

Foram entrevistados 22 participantes: oito cirurgiões-dentistas da APS – cinco de centros de saúde da família, três de centros de saúde, cinco do centro de especialidades odontológicas, dois da gestão/regulação e sete usuários.

A média de idade dos cirurgiões-dentistas (n=15) foi 45,6 anos. Da APS, a maioria era mulheres (n=7), e do CEO, homens (n=3). Em ambos os níveis, 100,0% eram especialistas e concursados. A maioria era graduada em instituição pública (seis da APS, quatro do CEO). O tempo de formado variou de 16 a 33 anos. A maioria dos cirurgiões-dentistas da APS e do CEO atuava entre 11 e 20 anos no SUS (n=10). Os dois da gerência eram mulheres e no cargo entre um e três anos.

Os usuários eram compostos em maioria por mulheres (n=5), aposentadas (n=4), casadas (n=4), com renda familiar mensal de até três salários mínimos (n=6), com média de idade de 68,2 anos e anos de estudo de 0-8 anos (n=3) e de 9-11 anos (n=3), tendo uma participante não informado. A maioria utilizava prótese em ambas arcadas (n=4), duas utilizavam apenas no maxilar, e uma não utilizava. O tempo de edentulismo total foi de 0-20 anos em ambas as arcadas (n=3), há ≥45 anos para arcada maxilar (n=3) e um usuário não sabia informar. Para a arcada mandibular, a maioria era desdentada parcial (n=4). O tempo de espera a partir do encaminhamento foi >12 e ≤24 meses (n=3), >24 e ≤36 meses (n=2) e de 0-12 meses (n=2). 

Observou-se a análise dos elementos constitutivos da rede de atenção à saúde bucal de Goiânia para tratamento com prótese dentária (Figura 1). O usuário (triângulo) percorre uma trajetória (setas ao longo da linha) desde a entrada na unidade de saúde até a instalação da prótese total no CEO. Quando o cirurgião-dentista da APS precisa encaminhar o usuário para prótese dentária, preenche a ficha de referência e a entrega ao usuário, que se direciona à recepção, onde a ficha é protocolada no sistema informatizado. A Central de Regulação de Vagas gerencia a lista de espera para a especialidade e o usuário; ao ser contemplado, ele é agendado para tratamento no CEO. 

**Figura 1:** Representação da rede de atenção à saúde bucal (RASB) para o tratamento com prótese dentária, a partir das falas dos cirurgiões-dentistas e usuários. Goiânia, 2019 (n=22)

Sobre esse processo, foi relatado o acionamento do Ministério Público por usuários para conseguir maior celeridade. Após acolher o usuário no CEO, o protesista preenche o laudo para autorização de procedimentos de alto custo/complexidade via meio eletrônico para justificar a solicitação da prótese. Os laboratórios regionais de prótese dentária são unidades de apoio para confecção da prótese. No CEO estadual, há o serviço de avaliação e da qualidade, que investiga as necessidades do usuário e o encaminha para outra especialidade, se necessário for. A ficha de contrarreferência é preenchida ao término do tratamento.

As categorias que emergiram das falas dos cirurgiões-dentistas da APS e dos CEO foram organizadas em comuns e em exclusivas (Tabela 1). Sobre o funcionamento do sistema de referência e de contrarreferência (categoria 1), o usuário é regulado no sistema informatizado e referenciado para os CEO. Alguns referenciam casos que consideram urgência com prioridade, porém desconhecem se são consideradas. 

**Tabela 1:** Categorias e subcategorias comuns e exclusivas da análise temática: falas dos cirurgiões-dentistas da Atenção Primária à Saúde (APS) e do centro de especialidades odontológicas (CEO). Goiânia, 2019 (n=13)

Categorias	Subcategorias	Principais falas
**Categorias e subcategorias comuns**		
1. Funcionamento do Sistema de Referência e Contrarreferência	1.1 Encaminhamento do usuário da APS para o CEO	1.1 “Os pacientes são regulados pelo município, através das unidades básicas para o Centro de Especialidades.” (cirurgião-dentista 1 – CEO).
	1.2 Tentativa de priorizar determinados casos	1.2 “[...] paciente que usa uma prótese que está traumatizando e não vai ficar sem prótese é uma prioridade, porque ele precisa e a que ele tem machuca a gente pede urgência, mas não sei se adianta.” (cirurgião-dentista 5 – APS).
2. Comunicação entre os cirurgiões-dentistas da atenção primária e secundária	2.1 Devolutiva da contrarreferência e divergências no uso desse mecanismo	2.1 “[...] a maior dificuldade que a gente tem é ver se tem resultado nosso encaminhamento porque a gente nunca teve um paciente que voltou [...]” (cirurgião-dentista 6 – APS). 2.1 “Sempre quando eu termino o tratamento eu faço a contrarreferência para atenção básica” (cirurgião-dentista 2 – CEO).
	2.2 Importância da comunicação/interação entre os profissionais	2.2 “Com certeza [seria interessante manter o contato], até pra gente acompanhar a evolução do tratamento desse paciente.” (cirurgião-dentista 1 – APS).
3. Percepção sobre a resolutividade das demandas protéticas	3.1 Restritas alternativas de tratamento protético no CEO	3.1 “Poderia ampliar o espaço físico, o número de pessoas que atende, oferecer tratamento com implantes dentários, próteses fixas, do tipo metalo-cerâmica, por exemplo, que aqui não tem.” (cirurgião-dentista 3 – CEO).
	3.2 Recursos limitados/congestionamento do CEO	3.2 “[...] precisaria aumentar a quantidade de laboratórios, de dentistas que atendem, protesistas, a oferta ainda é pequena em vista da demanda grande que existe.” (cirurgião-dentista 5 – APS). 3.2 “Os pacientes esperam muito tempo, muito tempo. Não é questão de seis meses, é de anos.” (cirurgião-dentista 2 – APS). 3.2 “[...] a gente teria uma demanda maior de pacientes se a gente tivesse mais profissionais, mais instrumentais, mais materiais, mais técnicos em laboratório.” (cirurgião-dentista 2 – CEO).
	3.3 Satisfação do usuário/atendimento às necessidades dos pacientes/disponibilidade do tratamento	3.3 “[...] na sua grande maioria, os pacientes que recebem prótese saem extremamente satisfeitos.” (cirurgião-dentista 4 – CEO).
	3.4 Complexidade do percurso entre os pontos da RASB	3.4 “A maior dificuldade é para o paciente conseguir chegar até aqui devido a essa peregrinação, ter que passar por uma unidade de saúde básica, depois, vai para um CEO [...]” (cirurgião-dentista 3 – CEO).
4. Papel da regulação	4.1 Gerenciamento e alocação das vagas	4.1 “Essencial. É com a regulação que a gente adota o critério de justiça e evita qualquer outro tipo de situação que não seja a do paciente regulado.” (cirurgião-dentista 5 – CEO).
	4.2 Dificuldade na compreensão do funcionamento da regulação	4.2 “[...] eu não sei como é que funciona a regulação.” (cirurgião-dentista 3 – APS).
**Categorias e subcategorias exclusivas**		
5. Percepção quanto ao papel da APS (cirurgião-dentista – APS)	5.1 Responsável pelo fluxo do usuário	5.1 “[...] o paciente chega, a gente identifica aquele problema e encaminha se necessário e o que a gente pode resolver aqui a gente faz.” (cirurgião-dentista 4).
	5.2 Provisão de atenção à saúde bucal	5.2 “[...] a gente faz orientações com relação à manutenção da prótese, higienização, cuidados com a prótese, no sentido de prevenção e promoção de saúde bucal [...]” (cirurgião-dentista 1).
6. O CEO no contexto da rede (cirurgião-dentista – CEO)	6.1 Profissionais especialistas, capacitados e integrados com as demais especialidades	6.1 “Todos fazemos curso de atualização. Procuramos nos dedicar muito, estudar e corrigir algumas falhas. Conversar com os profissionais, interagir com as especialidades.” (cirurgião-dentista 2).
	6.2 Efeitos positivos do tratamento com prótese total convencional	6.2 “[...] quando a gente consegue chamar, atender e concluir o atendimento é sempre muito positivo, porque a gente consegue dar uma nova qualidade de vida para o paciente.” (cirurgião-dentista 5).

Os cirurgiões-dentistas ponderaram como a comunicação entre eles (categoria 2) seria importante para acompanhar a trajetória do usuário, mencionaram a ausência da contrarreferência da especialidade de prótese dentária para a APS e como esta contribuiria na aquisição de informações. Os profissionais demonstraram suas percepções sobre os fatores que favorecem e dificultam a resolução das demandas protéticas na rede (categoria 3): restritas alternativas de tratamento protético, recursos materiais insuficientes (equipamentos, insumos e instrumental), além de excessiva demanda, que configuram um congestionamento no CEO. 

Mesmo quando a condição bucal era desfavorável, como na reabsorção óssea alveolar, os cirurgiões-dentistas demonstraram acreditar na resolução das necessidades dos desdentados totais. Foi relatada satisfação dos usuários com o tratamento no CEO e com o fato de a prótese total convencional ser assegurada. 

Na categoria 4, houve compreensão heterogênea sobre o papel da regulação de gerenciar as vagas e direcionar os usuários; alguns consideraram-na importante para a organização do sistema, enquanto outros aparentaram desconhecimento. Na categoria 5 (exclusiva da APS), os profissionais concordaram que a atenção primária à saúde cumpre a responsabilidade pelo fluxo do usuário, realizando o acolhimento, a avaliação e o encaminhamento para o CEO, além de assistência e ações de prevenção da saúde. Aspectos positivos sobre o tratamento no CEO (categoria 6 – exclusiva dos cirurgiões-dentistas do centro) foram elencados, como profissionais qualificados e disponibilidade de prótese total convencional.

Observaram-se as categorias e as subcategorias das falas das cirurgiões-dentistas da gestão/regulação (Tabela 2). Para elas, a regulação é importante para a rede (categoria 1), com o direcionamento imparcial das vagas, seguindo a ordem da lista e a análise individual dos casos, que podem ser considerados prioritários.

**Tabela 2:** Categorias e subcategorias da análise temática: falas das cirurgiãs-dentistas da gestão/regulação. Goiânia, 2019 (n=2)

Categorias	Subcategorias	Principais falas
**1. Atuação da regulação**	Nenhuma	1.1 “A gente analisa caso por caso (a História da Doença Atual de cada paciente). Os que são prioridade vão ocupar as vagas primeiro, os que não são, vão para o final da fila e a prioridade são dos pacientes mais antigos.” (cirurgião-dentista 1 – gestão/regulação).
**2. Gestão da saúde bucal no nível central da rede**	2.1 Intenção de ampliação das alternativas de prótese dentária pelo município	2.1 “Nós temos projetos para ofertar a prótese parcial removível também.” (cirurgião-dentista 2 – gestão/regulação).
	2.2 Uso das vagas da atenção especializada em saúde bucal por outros municípios	2.2 “Vem específico, tem duas para o interior e cinco para Goiânia [...]” (cirurgião-dentista 2 – gestão/regulação).
**3. Reconhecimento das limitações da rede**	3.1 Sobrecarga da atenção primária	3.1 “A grande demanda é porque a oferta é infinitamente menor do que a procura.” (cirurgião-dentista 1 – gestão/regulação).
	3.2 Dificuldades na priorização dos usuários	3.2 “Foi uma das dificuldades que a gente colocou aqui: priorizar paciente.” (cirurgião-dentista 1 – gestão/regulação).
	3.3 Presença parcial da equipe de saúde bucal na Estratégia de Saúde da Família	3.3 “[...] ainda não tem equipe de saúde bucal em 100% das equipes de Saúde da Família.” (cirurgião-dentista 2 – gestão/regulação).

A gestão demonstrou intenção de ampliar as modalidades de reabilitação, desde que a estrutura disponível opere em totalidade (categoria 2). Ela possui relação administrativa com municípios goianos, o que influencia na distribuição das vagas do CEO. Fatores limitantes (categoria 3) foram: a demanda pela APS excede à capacidade de oferta; as dificuldades em estabelecer parâmetros de priorização para prótese dentária; e o acesso à saúde bucal na Estratégia Saúde da Família está parcialmente implementado.

Observaram-se as categorias e as subcategorias das falas dos usuários (Tabela 3). De todos os encaminhados pela APS, dois acionaram o Ministério Público (categoria 1). A maioria mencionou morosidade para acessar o CEO, mas satisfação com o atendimento. Um participante demonstrou compreensão da alta demanda e da espera (categoria 2). Houve expectativa de maior satisfação com a nova prótese do que a atual, bem como de efeitos intra e interpessoais na retomada do sorriso, da autoestima e de um melhor convívio social (categoria 3).

**Tabela 3:** Categorias e subcategorias da análise temática: falas dos usuários. Goiânia, 2019 (n=7)

Categorias	Subcategorias	Principais falas
**1. Meio de acesso ao centro de especialidades odontológicas** (CEO)	1.1 Usuários referenciados para o CEO através da atenção primária	1.1 “Vim do CAIS, encaminhada pra cá.” (usuário 3).
	1.2 Acionamento do Ministério Público	1.2 “[...] tive que recorrer ao Ministério Público para vim parar aqui, senão, eu estava esperando em casa até hoje sem dente nenhum.” (usuário 1).
**2. Perspectiva sobre o tempo de espera para ser atendido no CEO**	2.1 A morosidade para ser contemplado com a vaga é superada pela satisfação com o atendimento	2.1 “Tive [dificuldade], pela demora. [...] Mas eu fiquei muito feliz quando eles me chamaram.” (usuário 5). “A demanda é muito grande. Então, tem que ser por espera mesmo [...]” (usuário 5).
**3. Expectativa dos usuários**	3.1 Expectativa positiva	3.1 “Espero que [a prótese] vai ficar melhor. Porque essa [prótese] que eu estou [...] está quebrada. Então, tenho certeza [de] que vai melhorar muito.” (usuário 5).
	3.2 Repercussões intra e interpessoais	3.2 “[...] poder sorrir, olhar no espelho e dar um sorriso e retomar a vida, a autoestima [...]. Vou poder frequentar ambiente, [...] porque conforme for o tipo da convivência, não teria como nem eu querer ter participação naquele meio [...]” (usuário 1).
**4. Percepção diante a satisfação**	4.1 Satisfação com o atendimento e recomendação do CEO	4.1 “Eu indico [o CEO]. O atendimento foi bom, pessoal competente e o atendimento foi bom, gostei. Eu aconselharia.” (usuário 2).
	4.2 In(satisfação) com a prótese total convencional	4.2 “A gente ouve falar que essa prótese debaixo é muito difícil de [se] acostumar, está mais ou menos dentro do esperado. Tem hora que a gente vai conversar, está querendo sair, tem que acudir.” (usuário 2). “Tudo foi bom. O dente novo está mais maravilhoso.” (usuário 6).
	4.3 Insatisfação com a ausência de alternativas	4.3 “Eu tinha um sonho de receber um implantezinho, uma prótese com quatro... pelo menos quatro. Dois na frente e dois mais atrás para firmar direito mas ainda não tem.” (usuário 2).
**5. Importância do Sistema Único de Saúde** (SUS)	5.1 Dependência do SUS	5.1 “[Se o CEO não existisse] Eu não tinha jeito de fazer nada. [...] Eu tenho uma dentadura mas já está muito velha, está com cinquenta anos.” (usuário 6).
	5.2 Noção do direito à saúde	5.2 “[Se o CEO não existisse] Provavelmente, eu ia recorrer em todos... depois que eu procurasse isso aqui tudo, fui no Ministério Público, de lá do Ministério Público, minha tendência era bater na porta da imprensa, na rede de jornalismo.” (usuário 1).

Os usuários expressaram satisfação e disseram que indicariam o CEO. Houve satisfação parcial com a retenção da prótese mandibular e insatisfação com a ausência de alternativas de tratamento, como implante dentário e prótese parcial removível (categoria 4). 

Constatou-se a importância do SUS no restabelecimento da saúde e no direito à saúde (categoria 5). Alguns não teriam condições para pagar pela prótese, e outros buscariam o serviço privado quando possível. Dois participantes transpareceram conhecer o direito à saúde garantido por lei ao relatarem que procurariam auxílio do Ministério Público diante da inexistência de um CEO.

No CEO estadual, ocorreram em média 24,5 instalações de prótese dentária/mês em 2016 e 27,16 em 2019. Nesse período, atuavam quatro protesistas (20 horas/semana). A meta era 120 próteses instaladas/mês para tal quantidade de profissionais. Porém, 22,6% da meta foi alcançada em 2019.

No CEO municipal, a média mensal foi 8,25 instalações de prótese dentária em 2016 e 2,66 em 2019. Em 2016, atuavam cinco protesistas, enquanto em 2019 atuavam quatro (20 horas/semana). A meta de atendimento diário por cirurgião-dentista foi de cinco pacientes, um exame (paciente novo) e quatro tratamentos (retorno). Em 2019, apenas 2,21% da meta foi alcançada.

Houve confirmação entre as fases da pesquisa no ajuste da integração de dados. Observou se que a rede cumpre o papel de acolhimento dos usuários na APS e de encaminhamento à atenção especializada, mas apresenta-se congestionada para o tratamento de prótese dentária. A indisponibilidade de recursos materiais, a escassez de alternativas de prótese e a morosidade para acessar um CEO contribuíram para a baixa resolutividade das demandas protéticas, em consonância com o desempenho aquém das metas para a instalação de prótese dentária.

## Discussão 

Este estudo evidenciou fragilidades na estrutura operacional da rede de atenção à saúde bucal de Goiânia, especialmente na oferta e na produção de prótese dentária. A percepção de usuários e de profissionais apontou limitações no acesso, na resolutividade e no cumprimento de metas, sobretudo na atenção especializada do SUS.

A Lei nº 14.572/2023, que institui a Política Nacional de Saúde Bucal no SUS, reforça o direito ao acesso universal, equânime e contínuo aos serviços de saúde bucal. A APS tem papel central nesse processo, sendo responsável pelo acolhimento, pela avaliação e pelo encaminhamento do paciente ao nível especializado, conforme também previsto na Política Nacional de Atenção Básica (15) e na diretriz da integralidade do cuidado. Ainda assim, a oferta de prótese dentária na APS permanece restrita, apesar de ser uma atribuição possível das equipes.

Neste estudo, constatou-se que a APS acolhe o usuário, realiza avaliação bucal, assistência e encaminhamento para o nível especializado do SUS, além de ações de prevenção (15). O acolhimento está relacionado à longitudinalidade e à satisfação na APS (23).

Apenas uma parte das equipes realiza procedimentos de prótese, sendo a cobertura ainda insuficiente (11,24). No Centro-Oeste, onde se localiza Goiânia, houve queda na realização de moldagens e de retornos (16), o que indicou retrocesso na oferta desses serviços. Embora os cirurgiões-dentistas possam realizar procedimentos de prótese dentária na APS, nem todas as equipes de saúde bucal no Brasil os fornecem (24), como neste estudo, em que a oferta da prótese está na atenção secundária. A fase qualitativa com equipes de Belo Horizonte em 2019 (24) encontrou que a oferta de prótese e do diagnóstico de câncer bucal influenciam a sua performance (24). 

A realização de procedimentos de prótese, inclusive de prótese parcial removível, necessita ser mais incorporada nos processos de trabalho das equipes da APS. Isso, apesar dos desafios, contribuiria para a integralidade do cuidado em saúde (25).

A cobertura da saúde bucal na Estratégia Saúde da Família foi 31% no município, abaixo do desejado. A ausência de contrarreferência – importante para a articulação entre os níveis de atenção do SUS – também é crítica, com o Centro-Oeste apresentando os piores índices do país (26).

O tempo de espera para atendimento especializado é longo, especialmente para prótese dentária, o que desestimula os usuários e compromete o cuidado. Embora os CEO contem com protesistas, a produtividade está aquém do esperado, muitas vezes devido à falta de insumos, equipamentos ou por sobrecarga de demanda. As metas de produção de prótese total não vêm sendo alcançadas, mesmo com parâmetros estabelecidos de atendimento clínico e laboratorial (22). 

Foi verificado baixo cumprimento de metas dos CEO para endodontia na região Sul (12). No Maranhão, foi constatado que a maioria dos CEO não cumpriu as metas para endodontia, cirurgia oral e periodontia (13). Grandes filas, acessibilidade geográfica prejudicada e ausência de materiais foram aspectos negativos relatados pelos usuários na resolutividade dos serviços de saúde (27). No Brasil, em 2012, os motivos de falta de atendimento dos CEO envolveram a falta de funcionamento de equipamentos, insumos e instrumental (26), o que reforça a necessidade de manutenções regulares e processos de compras bem-sucedidas.

A perda dentária sem reabilitação afeta diretamente a qualidade de vida do indivíduo (3). Felizmente, usuários demonstraram satisfação com o atendimento no CEO e relataram expectativas positivas quanto à prótese dentária, principalmente no resgate da autoestima e da funcionalidade, achados semelhantes à investigação em que os usuários expressaram satisfação com o CEO (29).

Apesar das limitações deste estudo – como a realização das entrevistas com usuários em um único CEO em funcionamento e um possível viés de gratidão –, os achados contribuem para o planejamento e a qualificação da rede de atenção à saúde bucal de Goiânia. Recomenda-se reforço da oferta de prótese na APS, inclusão da oferta de outros tipos de prótese, expansão das vagas mensais nos CEO, adequação do número de protesistas e de recursos materiais, além de melhoria da gestão da lista de espera e do sistema de referência e de contrarreferência.

Salienta-se que a prótese total é assegurada pela rede, com atendimento das necessidades dos usuários na APS e devido encaminhamento para um CEO, onde encontram-se profissionais que realizam o tratamento disponível sempre que possível. Os usuários demostraram satisfação e apontaram expectativas positivas para a prótese dentária, na melhoria da autoestima, das suas relações interpessoais e das suas atividades funcionais.

A efetivação da Política Nacional de Saúde Bucal requer articulação entre os níveis de atenção à saúde e garantia de estrutura adequada para assegurar o cuidado integral aos usuários do SUS. 

## Data Availability

Os dados utilizados na pesquisa estão disponíveis sob demanda aos autores, através do contato informado na seção Correspondência.
